# Supplementary material for: Asymmetric Contribution of a Selectivity Filter Gate in Triggering Inactivation of CaV1.3 Channels
Source: bioRxiv. 2023 Sep 23:2023.09.21.558864. Preprint. [Version 1] doi: 10.1101/2023.09.21.558864 (PMC10542529; doi:10.1101/2023.09.21.558864)
Supplement: Supplement 1 [file NIHPP2023.09.21.558864v1-supplement-1.pdf]

bioRxiv preprint doi: https://doi.org/10.1101/2023.09.21.558864; this version posted September 23, 2023. The copyright holder for this preprint (which was not certified by peer review) is the author/funder, who has granted bioRxiv a license to display the preprint in perpetuity. It is made available under aCC-BY 4.0 International license.

Supplementary Information

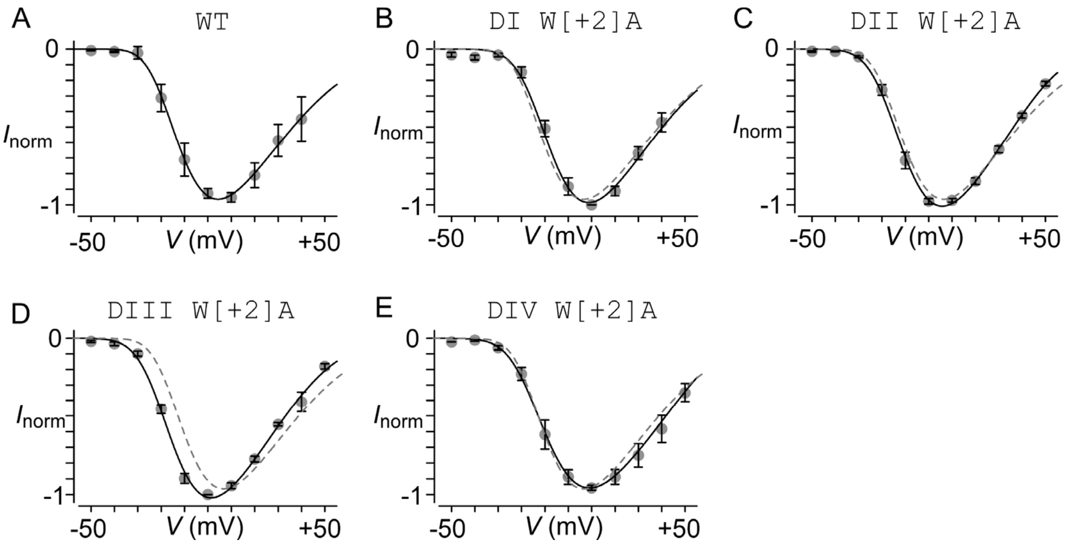

Figure S1. Cav1.3 DI-DIV SF mutations minimally perturb channel activation. (**A**) Normalized current voltage-relationship for WT Cav1.3. Each dot, mean ± s.e.m. n = 5 cells. (**B – E**) Normalized IV relations for DI/W[+2]A (panel B, n = 5 cells), DII/W[+2]A (panel C, n = 6 cells), DIII/W[+2]A (panel D, n = 5 cells), and DIV/W[+2]A (panel E, n = 7 cells) mutants. In all cases, the change in channel half-activation potential ($V_{1/2}$) was less than 5 mV.

bioRxiv preprint doi: https://doi.org/10.1101/2023.09.21.558864; this version posted September 23, 2023. The copyright holder for this preprint (which was not certified by peer review) is the author/funder, who has granted bioRxiv a license to display the preprint in perpetuity. It is made available under aCC-BY 4.0 International license.

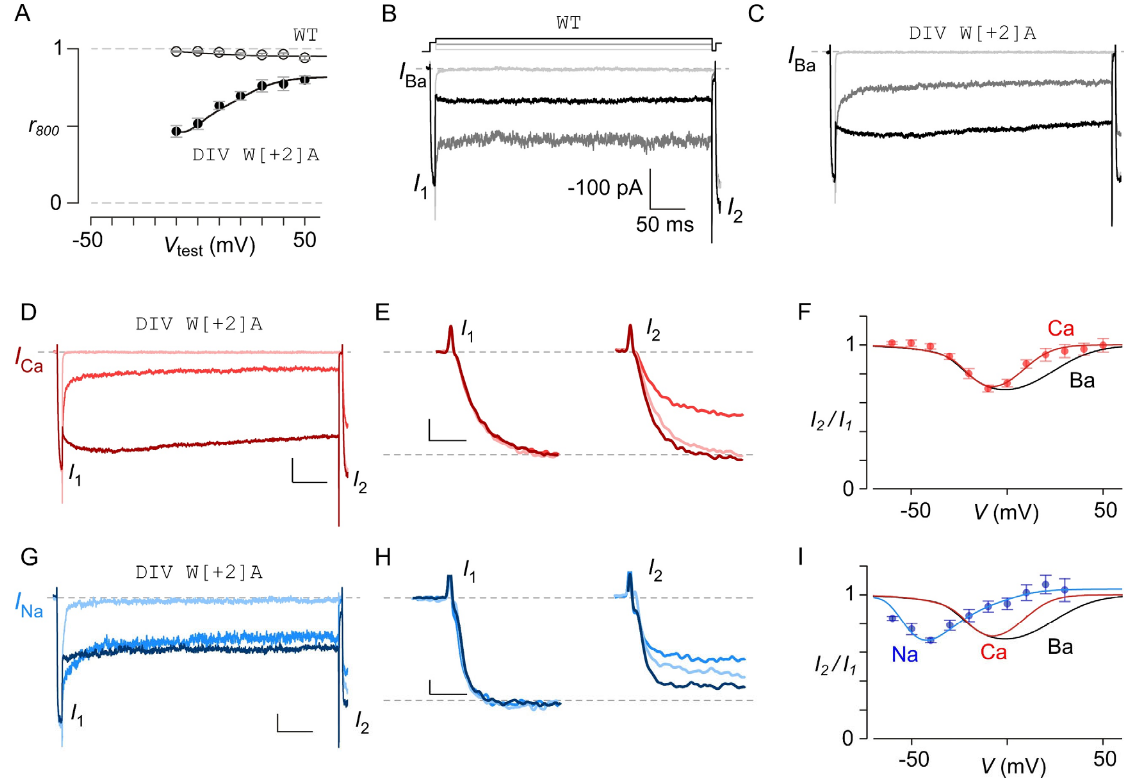

Figure S2. Extended Biophysical characterization of DIV W[+2]A mutation. (**A**) Population r800 data plotted as a function of test-pulse potential reveals reduced inactivation at higher voltages. Each dot, mean ± s.e.m. $n = 7$ cells. (**B**) A two-pulse protocol is used to dissect voltage-dependence of inactivation. A brief 15 ms prepulse to 10 mV quantifies available current prior to inactivation. Subsequently, a family of 800 ms voltage steps (test pulse) is used to evoke steady-state inactivation and a 15 ms postpulse is then used to quantify the current remaining following VDI. The extent of inactivation is quantified as the ratio of peak currents during post-pulse to pre-pulse. Exemplar currents at three different test pulse potentials (light gray, -50 mV; gray, 0 mV; and black +50 mV) are shown. Exemplar trace for wild-type Cav1.3. Further analysis in main text Fig. 2C, E. (**C**) Exemplar trace for DIV W[+2]A mutation. Further analysis in main text Fig. 2D, F. (**D – F**) VDI of DIV / W[+2]A probed in the presence of Ca^{2+} as charge carrier. Panel D, exemplar trace. Panel E, comparison of Ca^{2+} currents during pre-pulse (left, I_1) versus post-pulse (right, I_2). Note that the current magnitude during the post-pulse is maximally reduced at intermediate voltages. Panel F, population data shows U-shaped dependence of inactivation with Ca^{2+} as charge carrier (red). Black trace, relationship with Ba^{2+} as charge carrier reproduced from Fig. 2F. Each dot, mean ± s.e.m, $n = 8$ cells. (**G-I**) VDI of DIV / W[+2]A mutation with Na^{+} (but no divalents) as charge carrier. Format as in Panel D-F. $n = 7$ cells.
